# Supplementary material for: Osteocyte TSC1 promotes sclerostin secretion to restrain osteogenesis in mice
Source: Open Biol. 2019 May 15;9(5):180262. doi: 10.1098/rsob.180262 (PMC6544986; doi:10.1098/rsob.180262)

**Electronic supplementary material**

**Supplementary Materials and methods**

**Chromatin immunoprecipitation (ChIP) assay**

ChIP was carried out using a SimpleChIP® Plus Enzymatic Chromatin IP Kit (Cell Signaling Technology, #9005) according to the manufacturer’s. Immunoprecipitation was conducted with Histone H3K9ac antibody (Active Motif, 61251, 1:200) .Relative enrichment with -3602-3738- prime served as negative control. Enrichment of H3K9Ac on Sost promotor segments were analyzed in the previous study([Cohen-Kfir et al., 2011](#_ENREF_1)) and primers were listed in Supplementary Table S4.

Supplementary reference

Cohen-Kfir, E., Artsi, H., Levin, A., et al. (2011). Sirt1 is a regulator of bone mass and a repressor of Sost encoding for sclerostin, a bone formation inhibitor. Endocrinology *152*, 4514-4524.

Supplementary figure legends

Supplementary Figure S1.Generation of mice with osteocyte-specific deletion of TSC1.

(A) Schematic of deletion of TSC1 by *DMP1-*Cre-mediated recombination. This mutant carries a "floxed" allele of *Tsc1*, when combined with a mutant carrying DMP1-Cre recombinase gene, exons 17 and 18 of Tsc1 are deleted in osteocytes. (B) Genotyping the offspring after mating transgenic Cre and loxp mice. Original image of agarose gel electrophoresis. The expected band of mutant carrying a DMP1-Cre-recombinase gene is nearly 534 bp and floxed *Tsc1* allele is nearly 195 bp. M, molecular weight marker. *DMP1*^-^*Tsc1*^f/f^: control mice/DTCL, *DMP1*^+^*Tsc1*^f/f^: knockout mice/*Tsc1* CKO. (C) Osteocyte deletion of TSC1 was confirmed in cortical sections from Rosa-*DMP1* mice. Red signal indicates DMP1 expression in osteocytes. Boxed area was magnified on the right. Scale bar, 50 µm (n = 6)

Supplementary Figure S2 . General phenotypes of *Tsc1* CKO mice

(A) Body length and (B) weight in 10-week-old control and *Tsc1* CKO mice. No difference was detected in control and Tsc1 CKO mice (t test, *p* = 0.1235 and *p* = 0.0873, n = 6). ns, no significance. (C-D) Representative micro-computed tomography (micro-CT) 2D images of (C) vertebral column, (D) metaphyseal trabecular bone of 4-, 8-, and 12-week-old control and *Tsc1* CKO mice. Scale bar, 1 mm (n = 6).Data represent mean ± SD.* *P* < 0.05 ** *P* < 0.01, ****P* < 0.001 by Student’s t test.

Supplementary Figure S3 . Thickness of cortical bone in DTCL and *Tsc1* CKO mice

(A) H&E staining of cortical bone of femora from 4-, 8- and 12-week-old control and Tsc1 CKO mice. The boxed area was magnified in the panel below. The scale bar represents 100 µm and 50 µm (n = 6). (B-C) Histomorphometric measurements showed that (B) the mineralizing surface/bone surface (MS/BS, t test, *p* = 0.0087) and (C) bone formation rate (BFR, t test, *p* = 0.0003) of control mice were lower than that of Tsc1 CKO mice (n = 6). (D) mRNA expression of TSC1 in DTCL and Tsc1 CKO mice (t test, *p* = 0.0007). Data are represented as mean ± SD, ***p* < 0.01, and ****p* < 0.001.

Supplementary Figure S4 . TSC1 deletion induced H3K9 acetylation of the Sost promoter.

(A)CHIP-qPCR detected TSC1 binding to the Sost promotor region in MLO-Y4 with control or TSC1 shRNA lentivirus infection. (t test, -3602-3728, *p* = 0.1476; -761-844, *p* = 0.7348; -998-1115, *p* = 0.1037; -1245-1361, *p* = 0.0141;-1828-1940, *p* = 0.0209;

n = 3) .Data are represented as mean ± SD. * *p* < 0.05.


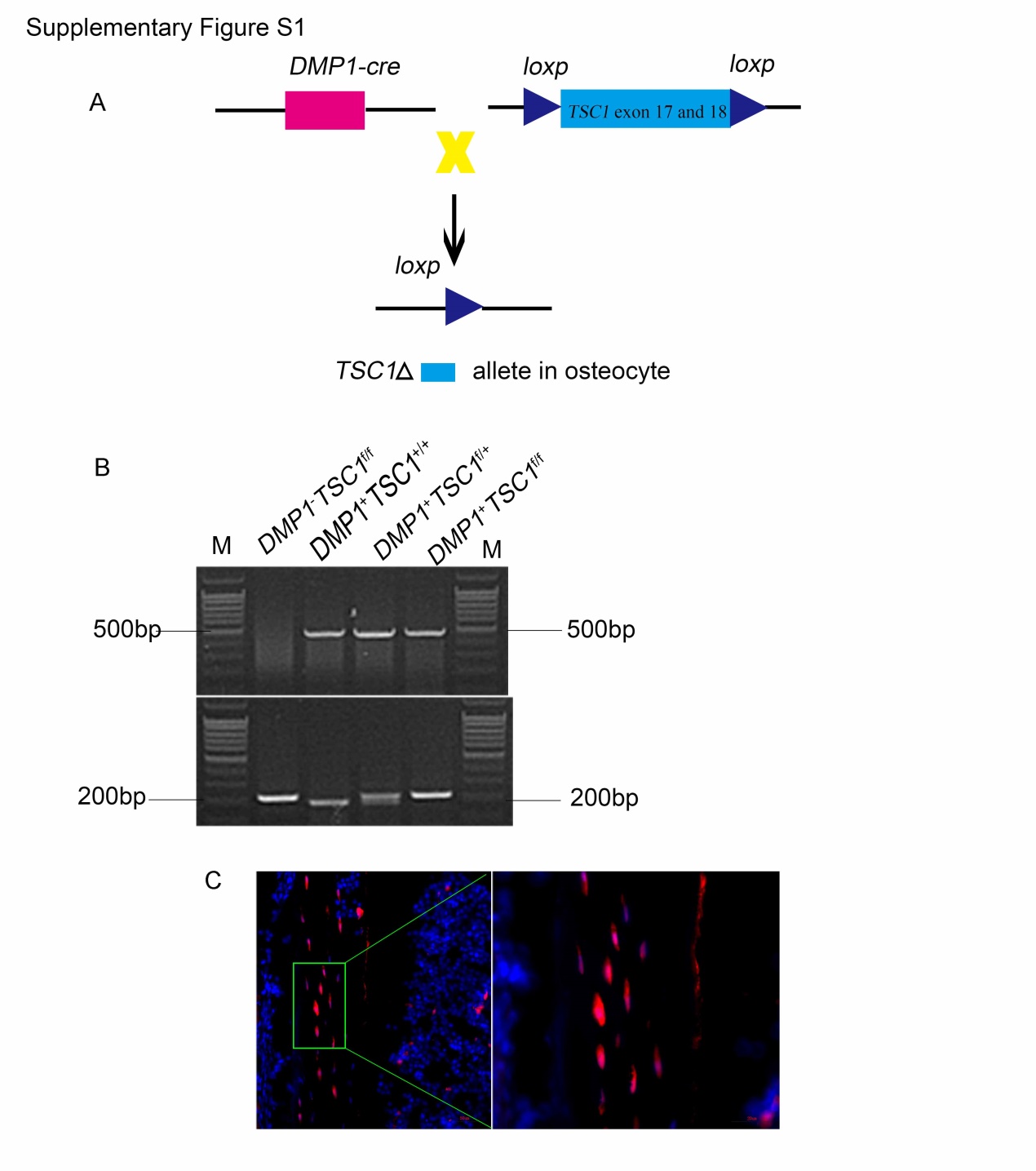


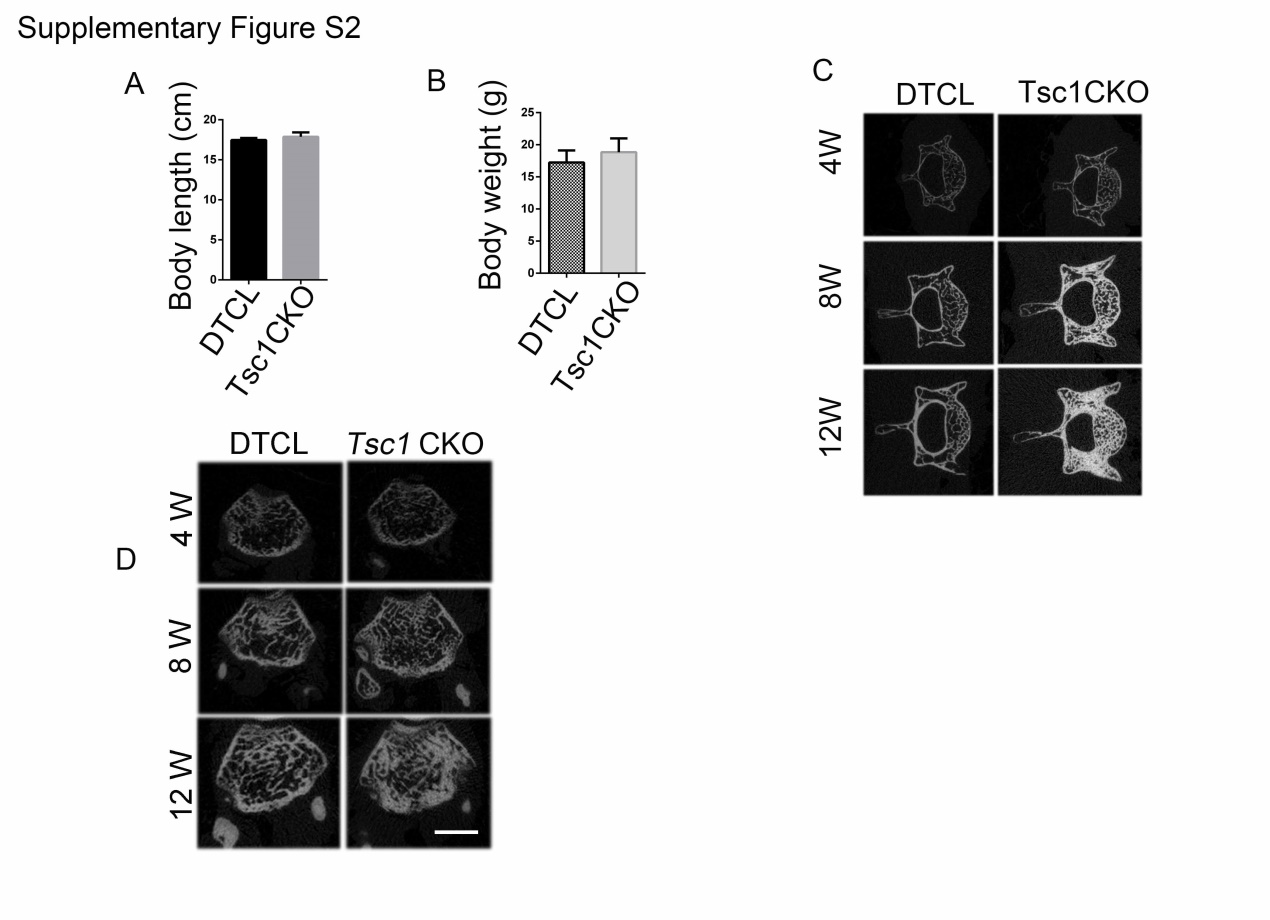


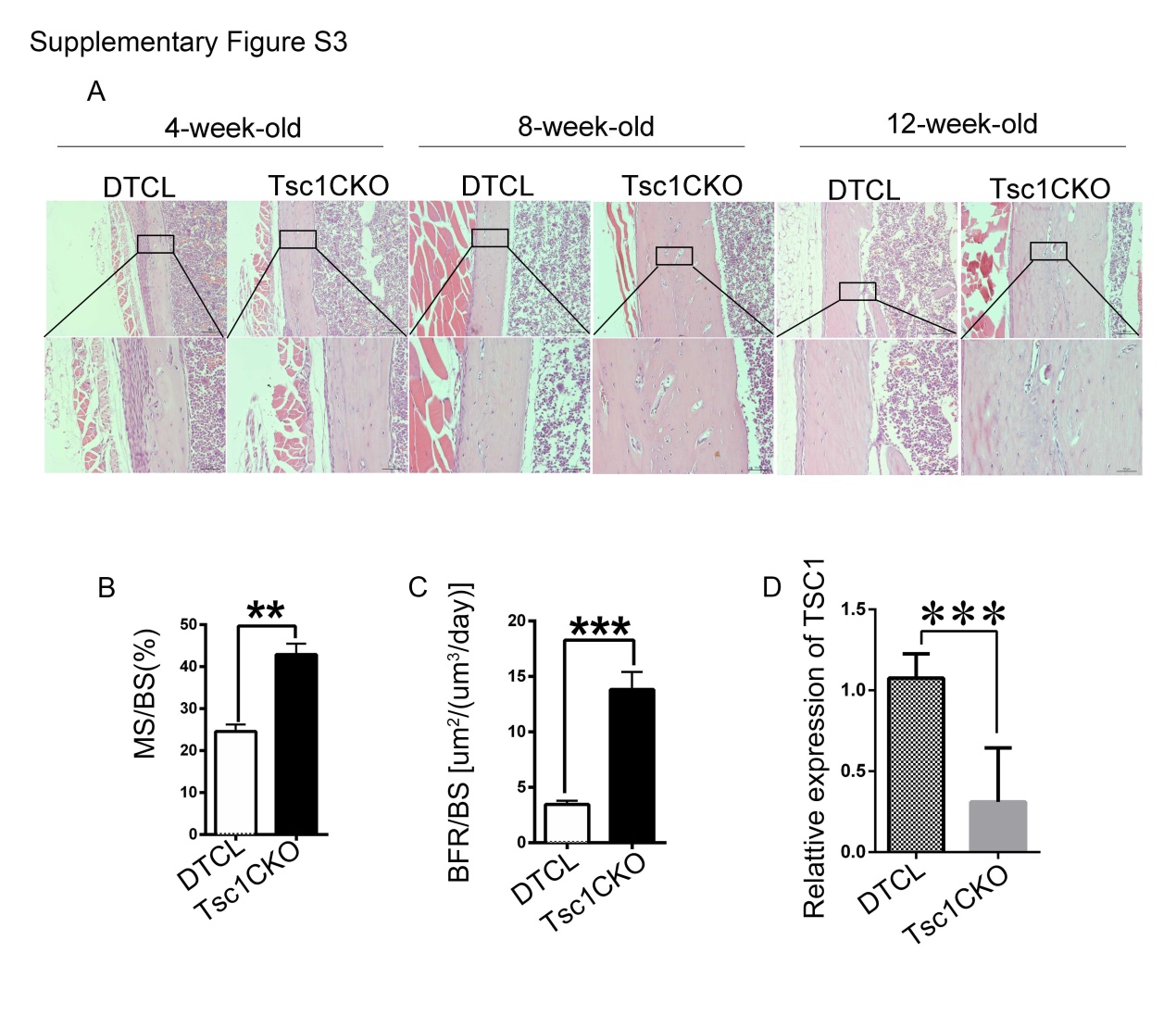


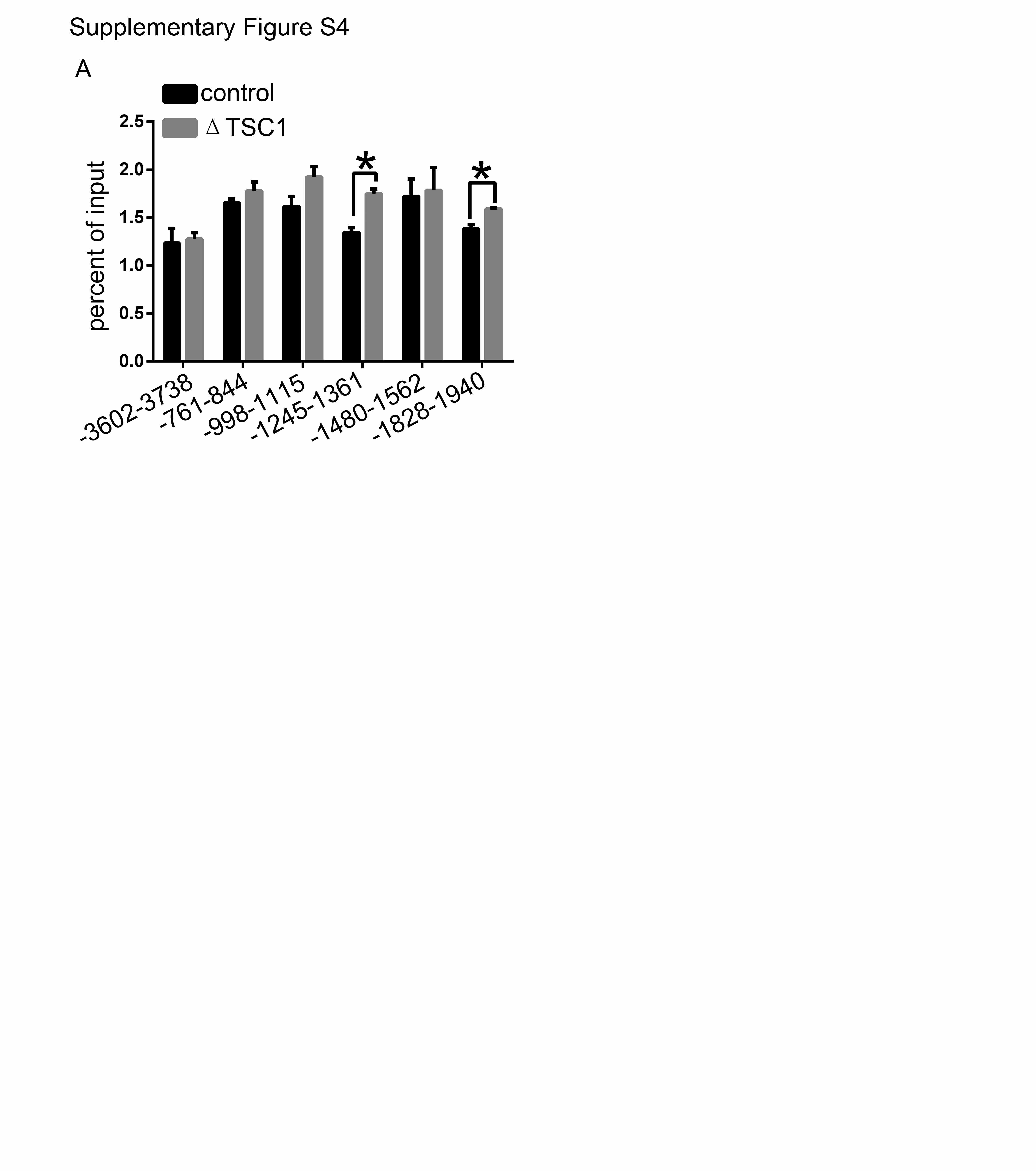

Supplement: Supplementary Figures [file rsob180262supp1.docx]
